# The moderating role of authenticity between experience economy and memory? The evidence from Qiong Opera

**DOI:** 10.3389/fpsyg.2022.1070690

**Published:** 2022-11-29

**Authors:** Yong Chai, Junli Na, TianCheng Ma, Ying Tang

**Affiliations:** ^1^School of Tourism, University of Sanya, Sanya, China; ^2^Ningxia Art Vocational College, Yinchuan, China; ^3^School of Business, Lingnan Normal University, Zhanjiang, China

**Keywords:** intangible cultural heritage, tourism experience, experience economy, memory, authenticity, behavior intention

## Abstract

Scholars have used the experience economy to analyze the behavior of tourists. However, in the field of intangible cultural heritage (ICH) tourism, the relationship between the experience economy and the behavior intention of tourists has not been studied. Scholars also point out that the relationship between the four dimensions of the experience economy is not static, and that aesthetic experience may be predictive of other dimensions. This study uses aesthetic experience as the starting point and constructs a theoretical model that includes the experience economy, the memories of ICH tourists, the perception of authenticity, and behavioral intentions. Qiong Opera, part of China’s national intangible cultural heritage, is used as a scenario in which to conduct empirical research. The results show that education, entertainment, and escape play a mediator role in the relationship between aesthetics and memory; memory plays a complete mediator role in the relationship between education, entertainment, escape, and behavioral intention; and authenticity plays a moderator role in the relationship between education, entertainment, escape, and memory. This study introduces the experience economy into ICH tourism. While expanding the application field of experience economy theory, it also provides theoretical and management inspiration for ICH tourism development.

## Introduction

Intangible cultural heritage (ICH) is an important part of tangible heritage and world heritage and an important manifestation of cultural diversity. However, current ICH protection and legacy issues in various countries are under tremendous pressure. The discourse around ICH has prioritized all areas that are intrinsically linked to human behaviors and perceptions (Giglitto et al., 2022). One of the main reasons is the lack of sufficient capital investment. With the general reduction in ICH protection funds, an increasing number of ICH managers regard tourism as a channel for generating finance (Esfehani and Albrecht, 2019). ICH tourism development is one of the important ways to alleviate the pressure on ICH protection funds (Tan et al., 2022). The deep integration of ICH and tourism means that ICH has become an important cultural tourism resource, and the status of ICH tourism products in the tourism industry is increasingly prominent (Esfehani and Albrecht, 2018; Yuan et al., 2022). Kirshenblatt-Gimblett (1998) identified that intangible cultural heritage has the potential to transform local communities into tourist destinations, and the economic benefit obtained can achieve sustainable development. WTO (2012) pointed out that tourism can promote the development of ICH’s protection. There is a need for research on ICH tourism to provide useful guidance for its sustainable development. ICH tourism can attract more tourists to a destination and promote local economic development.

Reisinger (1994) believed that cultural tourism is a special interest and an experiential form of tourism. Its main purpose is to seek out and participate in in-depth experiences with aesthetic, intellectual, emotional, or psychological properties. McKercher (2002) pointed out that in-depth experience is an integral part of cultural tourism. As ICH is a resource for cultural tourism, it has the characteristics of this industry and must be developed in accordance with its guidelines (York et al., 2021). In other words, ICH tourism must attract tourists’ continuous attention and provide them with a real tourism experience (Guo et al., 2022). Chen (2022) findings suggest that intangible cultural heritage tourism can enhance the cultural experience of tourists. Katelieva et al. (2019) confirmed that for most tourists, learning and experiencing local customs is more important than understanding the origin or authenticity of the customs. However, because many tourists do not have a local cultural background and traditional knowledge, it can be difficult for them to appreciate and experience intangible cultural heritage (Esfehani and Albrecht, 2019). As the results of previous studies show, ICH tourism relies on the experience economy (Dean, 1976).

The experience economy is an extension of the service economy and is the fourth type of economy after the agricultural, industrial and service economies, emphasizing the emotional satisfaction of the customer and the psychological experience of the customer when the consumer behavior occurs (Sundbo, 2021). The experience economy has been researched in rural tourism (Loureiro, 2014), festival tourism (Tom Dieck et al., 2018), gourmet tourism (Shen, 2021), religious tourism (Su et al., 2020), and the hospitality industry, but it has not been investigated in the ICH tourism field. Therefore, exploring the experience of tourists in ICH tourism from the perspective of the experience economy has particular significance in expanding the theoretical application of the experience economy. In previous research, the four dimensions of the experience economy all emerged as a side-by-side structure (Zhang et al., 2021). However, the study by Tom Dieck et al. (2018) shows that the structure of the experience economy has changed. The authors found that in the experience economy, esthetics is the most important factor and the antecedent variable affecting education, entertainment, and escape. Relatively few studies have used this structure. Therefore, this study conducted an empirical test of the structure to verify Tom Dieck et al. (2018) results. Authenticity is also a topic of great concern in cultural tourism. However, the relationship between the experience economy and authenticity has yet to arouse widespread interest among scholars. In empirical research, only Kirillova et al. (2017) conducted a preliminary discussion on this, but the structure they adopted was different from the four-dimensional structure adopted in this research. Therefore, in the emerging ICH tourism, whether their relationship is consistent with Kirillova et al. (2017) research, and whether using different structures will lead to differences in the results, are worthy of further discussion.

This paper is divided into six parts. The first part is the introduction, which focuses on the background, purpose and significance of this study; the second part is the theoretical basis and hypothesis building; the third part describes the methodology used in this study, the fourth part is the results of the study, the fifth part is the discussion and conclusion, and the last part is the limitations of this study and future research outlook.

## Literature review

### Experience economy

Pine and Gilmore (1998) identified the four stages of economic development. The first is the commodity exchange stage; the second is the commercial economy stage; the third is the service economy stage; and the fourth is the current experience economy stage. The biggest difference between the experience economy and the service economy is that the service economy only provides intangible or customized services, and the experience economy aims to offer an unforgettable experience (Pine et al., 1999). Pine et al. (1999) proposed four dimensions of the experience economy: education, esthetics, entertainment, and escape. The authors also believe that the degree of customer participation is different, and that the experience itself is different. Oh et al. (2007) developed a set of scales, based on the concept and framework of Pine et al. (1999), to measure visitors’ perception of the dining experience. Tom Dieck et al. (2018) research further modified Oh et al. (2007) scale to investigate the experience of tourists participating in festival activities. Previous studies have shown that tourism is an early example of the experience economy. A travel experience is a one-of-a-kind experience that visitors have, and it includes emotional components. The experience economy is still garnering attention in tourist research, according to the extant literature (Alexiou, 2019; Hwang and Lee, 2019; Zhang et al., 2021), showing that the experience economy has important value in the field of tourism.

According to earlier research, the experience economy connotations of the four relatively independent characteristics are taken into account as a whole (Zhang et al., 2021). However, these four independent dimensions seem to have different effects on tourists. Hosany and Witham (2010) conducted a study on the impact of luxury cruise tourists’ travel experiences on their willingness to recommend the cruise experience to others and found that aesthetic experiences have the greatest impact. Mykletun and Rumba (2014) found similar results. They believe that esthetics is the most important driving force in the experience economy. This result was also confirmed by Oh et al. (2007). However, Tom Dieck et al. (2018) study used theoretical derivation and showed that esthetics is an antecedent variable for education, entertainment, and escape. Their empirical research involved the use by tourists of augmented reality (AR) devices to participate in festival experiences. Their assumption has been shown to be true. It is thus clear that the architecture of the experience economy is not static and that different architectures may have to be used in different studies (Hwang et al., 2022). The experience economy is still a key topic of interest in the field of tourism, after all, it has direct reference value for tourism development, marketing and management, and provides more possible references for other scholars in subsequent studies (Zhang et al., 2021).

### Experience economy and memory

According to Pine et al. (1999) definition, the experience economy is an unforgettable special experience. Memory occupies an important position in the experience economy and plays an important role in tourism. Tourists’ activities are a means of gaining experiences, either positive or negative (Oh et al., 2007). Kahneman (2011) showed that tourism creates memories for tourists. Vickers (2017) and Lai et al. (2021) found that tourists’ memories of past pleasant travel experiences can enhance their emotions toward a destination.

There have been many empirical studies on the experience economy and memory. For example, Lai et al. (2021) combining experience economy theory and perceived value theory to explore electronic word-of-mouth in food tourism. Leung et al. (2022) explored the relationship between tourists’ VR tourism experiences on behavioral intentions in the context of COVID-19, using an experience economy perspective. Loureiro (2014) used rural tourism as the background to study the relationship between the experience economy and memory, entertainment, arousal, local attachment, and behavioral intentions, finding that the experience economy has a positive effect on memory. Among the four dimensions of the physical examination economy, the effect of esthetics is the greatest. Kastenholz et al. (2018) used rural tourism as the background to study the relationship between the experience economy and arousal, memory, and satisfaction. The results found that aesthetic experience has the greatest impact on memory. Oh et al. (2007) research on customers’ accommodation and dining experiences found that the effects of evasion and entertainment on memory are relatively weak in all dimensions of the experience economy. In Tom Dieck et al. (2018) study, only the direct relationship between education, entertainment, escape, and memory is discussed. All three have a positive effect on memory.

### Memory and behavior intention

Richard (1997) believed that behavioral intention refers to the subjective probability of people performing a certain behavior. The study by Zeithaml et al. (1996) further subdivided behavioral intentions, including the following: (1) Make a positive evaluation of the products purchased or services received; (2) Buy the product or service again; (3) Recommend the product or service to your relatives and friends; (4) Will to bear more costs for the product or service; (5) Loyalty to the enterprise that provides the product or service. In the tourism industry, scholars’ research on tourists’ behavioral intentions focuses on willingness to recommend and to revisit (Sharma and Nayak, 2018; Chen, 2022). Tourism businesses are also aware that tourists’ behavioral intentions are critical to the growth of their businesses (Pandža Bajs, 2015; York et al., 2021). This has a positive effect on their product marketing and brand image building (Zhang and Lee, 2022). Therefore, it is necessary to explore the behavioral intention of tourists in the marketing and management of tourism enterprises. In the existing literature, research on behavioral intentions mainly focuses on satisfaction (Hultman et al., 2015; Tom Dieck et al., 2018), perceived value (Pandža Bajs, 2015), experience quality (Altunel and Erkurt, 2015), memory (Wixom and Todd, 2005), authenticity (Castéran and Roederer, 2013), and other related variables.

Many scholars found that tourists’ memories have a positive effect on their behavioral intentions (Martin, 2010; Ding and Hung, 2021; Horng and Hsu, 2021). As Kim et al. (2022) finds, tourists’ active destination memory will increase the likelihood of first-time visitors returning, generating a favorable reputation for the place. Loureiro (2014) study of tourists’ experiences in rural tourism finds that memory has a positive effect on tourists’ behavioral intentions. Ali et al., 2014 study of tourists visiting Malaysia for vacations also finds that good memories enhance tourists’ loyalty to hotels, a result reconfirmed in the study of creative tourists by Ali et al. (2016).

### Mediating effect

In the new experience economy structure, esthetic experience has become the antecedent variable of educational experience, entertainment experience, and escape experience. Memory is the result variable of esthetic experience, educational experience, entertainment experience, and escape experience, respectively. In other words, educational experience, entertainment experience, and escape experience may have a mediating effect in the relationship between esthetic experience and memory. Lai et al. (2019) study of Chengdu cuisine, the new experience economy architecture was used and the mediating roles of educational experience, entertainment experience, and escape experiences between aesthetic experience and memory, and aesthetic experience and satisfaction, respectively, were confirmed. In addition，in a study by Horng and Hsu (2021), the mediating role of entertainment experience in the relationship between aesthetic experience and memory was confirmed. Therefore, this study proposes the following hypotheses:

*H1a*: Educational experience plays a mediating role in the relationship between esthetic experience and memory.

*H1b*: Entertainment experience plays a mediating role in the relationship between esthetic experience and memory.

*H1c*: Escape experience plays a mediating role in the relationship between esthetic experience and memory.

Based on the discussion of previous studies presented, educational experience, entertainment experience, and escape experience are the antecedent variables of memory, and behavior is the outcome variable of memory. That is, memory may have a mediating effect in the relationship between educational experience and behavior, between entertainment experience and behavior, and between escape experience and behavior. Loureiro (2014) research and the study by Tom Dieck et al. (2018) also explore the relationship between the experience economy and memory, memory and behavioral intention. Unfortunately, those studies did not explore the mediating role of memory in the relationship between experiencing economic and behavioral intentions. Some scholars found that the experience economy is an external stimulus, which can cause tourists to have emotions, which, in turn, have an impact on tourists’ behavioral intentions (e.g., Anderson and Shimizu, 2007; Ballantyne et al., 2011). Travel memory is a strong expression of emotion. Therefore, this study proposes the following hypotheses:

*H2a*: Memory plays a mediating role in the relationship between educational experience and behavior.

*H2b*: Memory plays a mediating role in the relationship between entertainment experience and behavior.

*H3c*: Memory plays a mediating role in the relationship between escape experience and behavior.

### Moderating effect

To understand the feelings of tourists in the process of heritage tourism (Mac Cannell, 1973) introduced the concept of authenticity into the field of tourism research. Tourist authenticity includes objective authenticity, construction authenticity, existence authenticity, and stage authenticity (Wong et al., 2018). The current research on authenticity typology is not mature enough to facilitate the study of authenticity. Wong et al. (2018) regard the authenticity perception of tourists as a combination of subjective and objective effects. Authenticity has become an important criterion for whether tourists’ experiences are meaningful (Lu et al., 2015). Su et al. (2020) believe that the experience of authenticity is extremely important in tourism, especially heritage tourism, and authenticity has become one of the important incentives to attract tourists. Duan and Marafa (2019) conducted a study of tourists in theme parks and found that most of the interviewees attached great importance to the perception of authenticity in the experience economy. Fridgen (1984) believes that the environment plays an important role in the experience of tourists, and it will directly affect the formation of tourists’ good memories. This environment includes tangible and intangible elements, and tourists’ perception of authenticity is an important intangible factor. Manthiou et al. (2018) research found that authenticity helps to enhance tourists’ memories. As existence authenticity is also a kind of authenticity, this study agrees with Wong et al. (2018) simplified concept of authenticity. The role of authenticity in enhancing memory has been verified by many scholars in the field of tourism research (Kim, 2021). Therefore, this study proposes the following hypotheses:

*H3a*: Authenticity plays a moderating role in the relationship between educational experience and memory.

*H3b*: Authenticity plays a moderating role in the relationship between entertainment experience and memory.

*H3a*: Authenticity plays a moderating role in the relationship between escape experience and memory.

**Figure 1:**
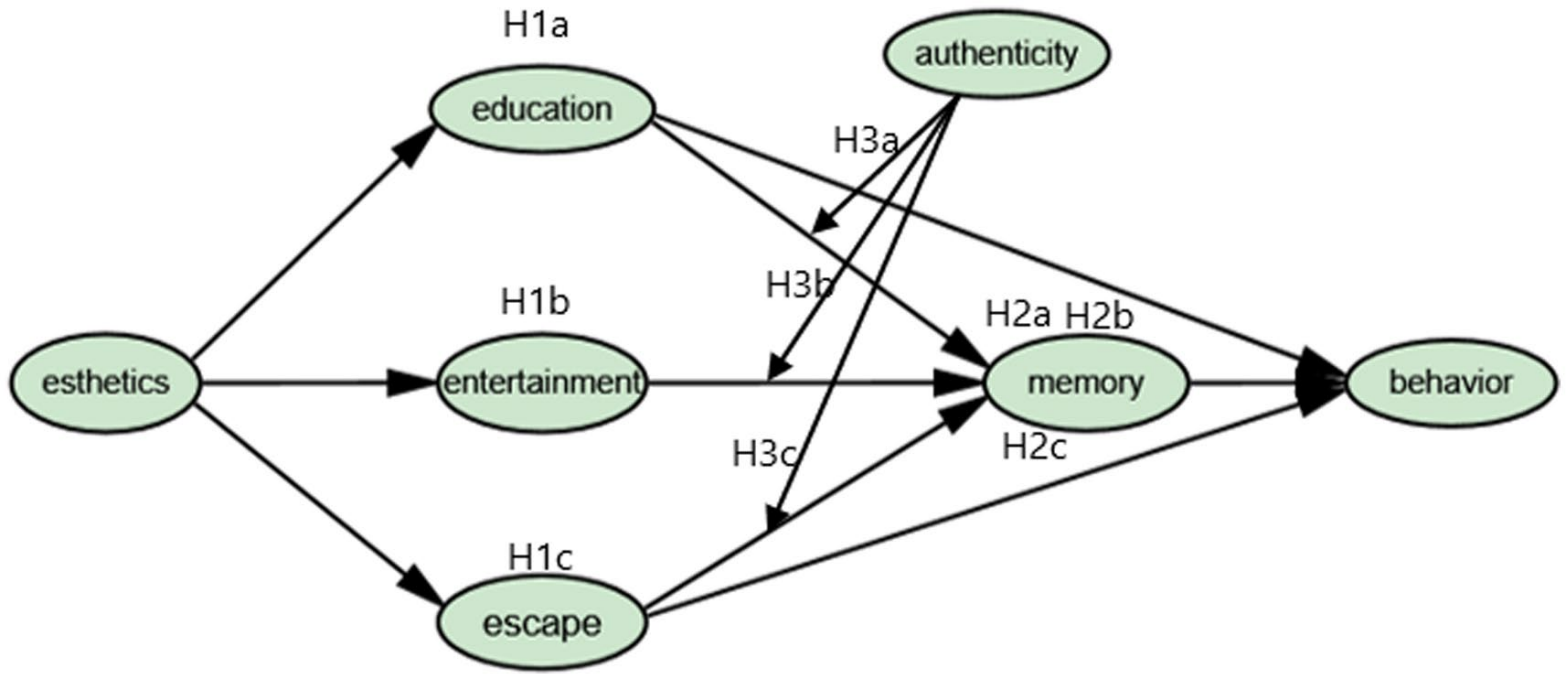
The research model.

Based on the above research hypotheses, this study constructed a theoretical framework, as shown in Figure 1 below.

## Materials and methods

### Research subject

**Figure 2:**
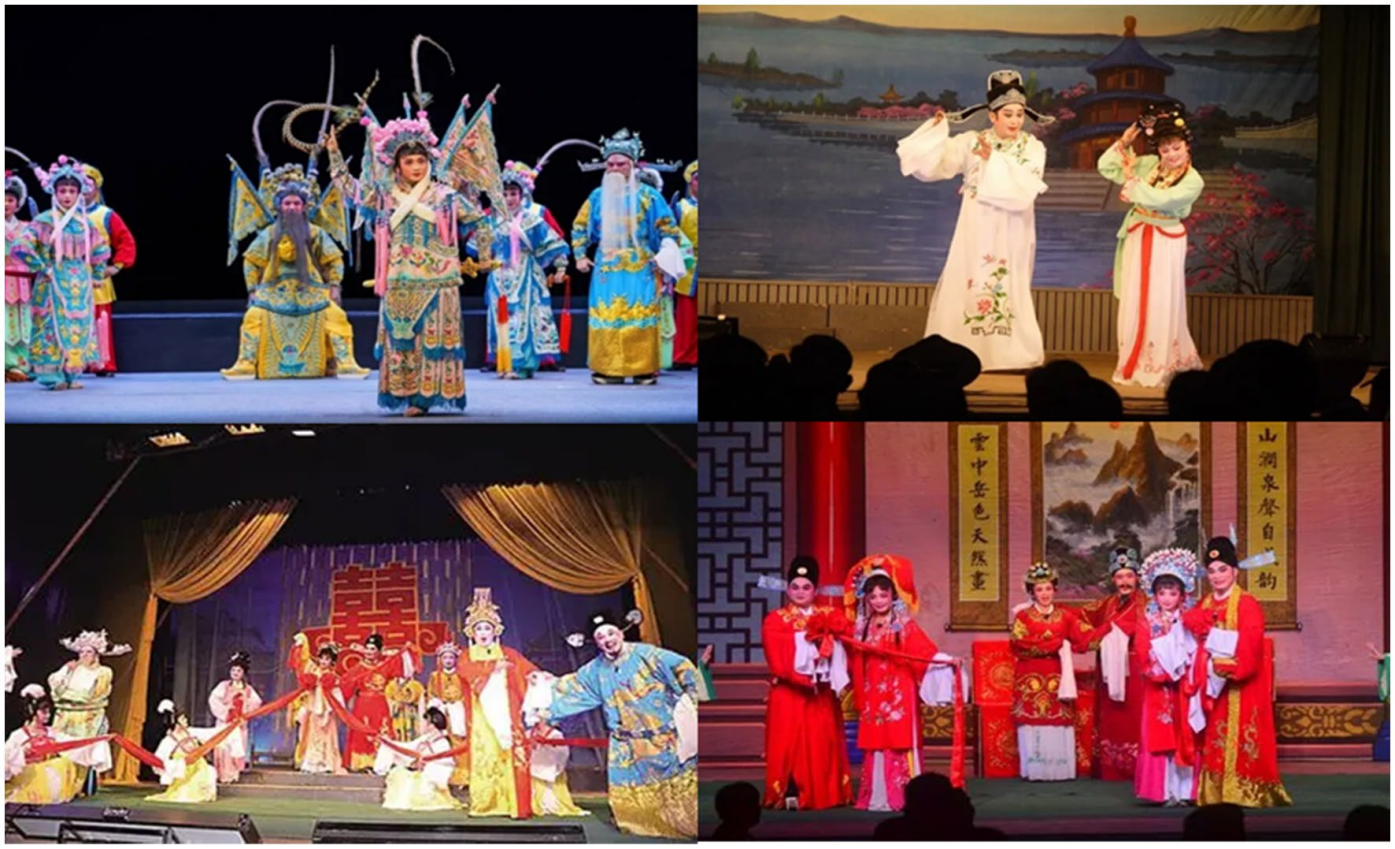
The Qiong Opera performance. Reproduced with permission from Qiong Opera.

Qiong Opera is the only local opera in Hainan Province. It has been called “Tuxi Opera” or “Hainan Opera” in the Qing Dynasty, “Zhai” in Qiongshan and Haikou, and “Qiongzhou Opera” and “Qiong Yin” by overseas Chinese. The name “Qiong Opera” first appeared in writing in 1936. Since then, this name has become widespread and has been used up to the present. Qiong Opera is a local opera culture developed and inherited by the people of Hainan from generation to generation. It has had an important impact on the history and development of Hainan and has important research value in anthropology, folklore, regional cultural studies, and the history of international cultural exchanges. It has been invited to perform in England, France, Italy, Japan, the United States, Germany, and many other countries. Images from performances are shown in Figure 2. At its peak, there were more than 50 Qiong Opera troupes in Hainan Province. However, nowadays, most of the actors and heirs to the tradition are old. Many families have much better incomes than before, and they are reluctant to allow their children to join the troupe to learn to perform. Qiong Opera risks not having performers, and the survival of the tradition is under severe pressure. However, there has been continuous advancement and deepening of the national cultural and tourism integration policy, and Qiong Opera has attracted many tourists. Many travel agencies recommend Qiong Opera as an entertainment attraction for tourists. The troupe has also taken the initiative of visiting universities and going on tour abroad to attract bigger audiences and to engage in a process of reinvigoration.

### Measurement

This study used self-report questionnaires to survey tourists who participated in the Qiong Opera experience. The questionnaire used a 7-point Likert scale (1 = strongly disagree, 7 = strongly agree), and consisted of five parts (see Table 1). The first part involved the experience economy, including four sub-variables, a total of 14 items, from Tom Dieck et al. (2018). The second part is memory, including four items, from Pine et al. (1999). The third part is authenticity, including four items from Lu et al. (2015). The fourth part involves behavioral intention, including three items from Jin et al. (2015). The fifth part covers demographics.

**Table 1:** Reliability and convergent validity of the constructs (*n* = 358).

Constructs/Items	Factor loading	*T*-value	CR	AVE
**Escape** (Cronbach’s alpha = 0.891)			0.892	0.673
I felt I played a different character when experiencing the Qiong opera	0.787			
I felt like I was living in a different time or place	0.796	16.046		
The Qiong opera experience let me imagine being someone else	0.843	17.207		
I completely escaped from reality	0.853	17.433		
**Authenticity** (Cronbach’s alpha = 0.892)			0.892	0.675
The Qiong opera is well preserved	0.866			
The Qiong opera reflects the true portrayal of ancient times	0.796	17.919		
The Qiong opera presents local history and culture very well	0.782	17.461		
The Qiong opera arouses feelings of authentic history and culture	0.838	19.335		
**Education** (Cronbach’s alpha = 0.818)			0.820	0.603
I learned something new during the Qiong opera experience	0.799			
The experience made me more knowledgeable	0.709	13.298		
It was a real learning experience	0.818	15.279		
**Memory** (Cronbach’s alpha = 0.825)			0.827	0.614
I will have wonderful memories about this Qiong opera experience	0.751			
I will not forget my experience of this Qiong opera experience	0.771	14.497		
I will remember many positive things about this Qiong opera experience	0.826	15.583		
**Behavior** (Cronbach’s alpha = 0.833)			0.832	0.623
I would like to re-experience this Qiong opera in the future	0.817			
I would recommend this Qiong opera to my friends or other acquaintances	0.758	14.152		
I want to tell other people positive things about this Qiong opera	0.793	14.674		
**Esthetics** (Cronbach’s alpha = 0.809)			0.809	0.587
The Qiong opera experience was very attractive	0.711			
The Qiong opera experience was very pleasant	0.749	12.334		
I felt a real sense of harmony	0.834	13.045		
**Entertainment** (Cronbach’s alpha = 0.857)			0.857	0.667
The Qiong opera experience was amusing	0.825			
The Qiong opera experience was entertaining	0.776	15.766		
The Qiong opera experience was fun	0.848	17.347		

CR, composite reliability; AVE, average variance extracted.

### Data collection

This research conducted a survey of tourists participating in the Qiong Opera experience at the ICH Heritage Center of Haikou Qiong Opera from October 1 to October 10, 2021. During the period, there were 20 performances, and each performance was attended by about 200 visitors. After each performance, 20 questionnaires were distributed. A total of 400 copies were distributed, and 369 were returned. Eleven of the questionnaires were incomplete and were eliminated. There were 358 valid questionnaires, and the effective recovery rate was 89.5%. Among the responders, 53.9% were male, and 46.1% were female. The main age groups were 18–25 years (*n* = 102 and 28.5%), 26–35 years (*n* = 87 and 24.3%), 36–45 years (*n* = 110 and 30.7%), and above 46 years (*n* = 59 and 16.5). The majority of the respondents had a high school diploma (*n* = 98 or 27.4%), a college diploma (*n* = 97 or 27.1%), or a bachelor’s degree (*n* = 110 or 30.7%). The personal monthly income was between 3,000 and 7,000 RMB (*n* = 223 or 62.3%). There were first-time visitors (*n* = 196 or 54.7%) and repeat visitors (*n* = 162 or 45.3%).

## Empirical results

SPSS24 was used to perform a quality inspection on the data set, and it showed that the kurtosis and skewness of the data meet the requirements, and there are no missing values or abnormal values. Therefore, the data set was suitable for the next analysis. At the same time, the single factor detection method was used to detect the common method variation of the data. The cumulative explanatory variance of the first variable after exploratory factor analysis was 42.143%, which did not exceed the recommended 50%. Therefore, in this study, the common method variation is acceptable (Harman, 1976). Using variance inflation factor (VIF) to detect the multicollinearity of each variable, it was found that all VIFs did not exceed 3. This shows that the problem of multicollinearity is not serious in this study and can be ignored. Then, the reliability test of each variable was carried out. Table 1 shows that the reliability (Cronbach’s α) of each variable is between 0.809 and 0.891, which meets the recommended standard recommended of 0.7 (Hair et al., 2010). This shows that the data in this study are of good quality.

### Measure model

In this study, AMOS24 was used to perform confirmatory factor analysis on the data. The model fit reached the recommended value (Hair et al., 2010); see Table 2-CFA. The factor loadings of all variables are between 0.716 and 0.853, thereby reaching the recommended standard of above 0.7 (Hair et al., 2010). The composition reliability of each variable is between 0.810 and 0.892, all of which meet the recommended standard of greater than 0.7; that is, each variable has good internal consistency (Hair et al., 2016); see Table 1. In this study, average variance extracted (AVE) was used to evaluate the convergent validity of each variable. Table 1 shows that the AVE of each variable is between 0.587 and 0.673, all of which meet the recommended standard of above 0.5 (Hair et al., 2014). Fornell and Larcker (1981) set out that each variable’s AVE square root should be greater than its Pearson correlation coefficient with other variables before it can be considered as having discriminative validity. Table 3 shows that the AVE square root of all variables is greater than its correlation coefficient with other variables. Therefore, the scale has good distinguishing validity.

**Table 2:** Results of the model fit measure.

Index	Chi-square	df	Chi-square/df	GFI	AGFI	RMSEA	NFI	RFI	CFI	IFI	TLI
CFA	286.516	209	1.371	0.937	0.917	0.032	0.941	0.929	0.983	0.983	0.980
Structure	341.333	142	2.404	0.899	0.865	0.063	0.911	0.893	0.946	0.946	0.935
Fitted value	——	——	<3	>0.9	>0.9	<0.08	>0.9	>0.9	>0.9	>0.9	>0.9

**Table 3:** Correlation and discriminant validity.

		Mean	SD	1	2	3	4	5	6	7
**1**	Escape	5.153	1.072	0.820						
**2**	Authenticity	5.120	1.410	0.395	**0.821**					
**3**	Education	5.288	0.955	0.658	0.348	**0.777**				
**4**	Memory	5.341	0.955	0.725	0.502	0.771	**0.784**			
**5**	Esthetics	5.270	1.290	0.463	0.329	0.469	0.686	**0.766**		
**6**	Entertainment	5.261	1.115	0.576	0.566	0.552	0.708	0.481	**0.817**	
**7**	Behavior	5.563	0.974	0.468	0.195	0.486	0.620	0.473	0.365	**0.790**

The bold fonts are the square roots of each AVE.

### Structural model

**Figure 3:**
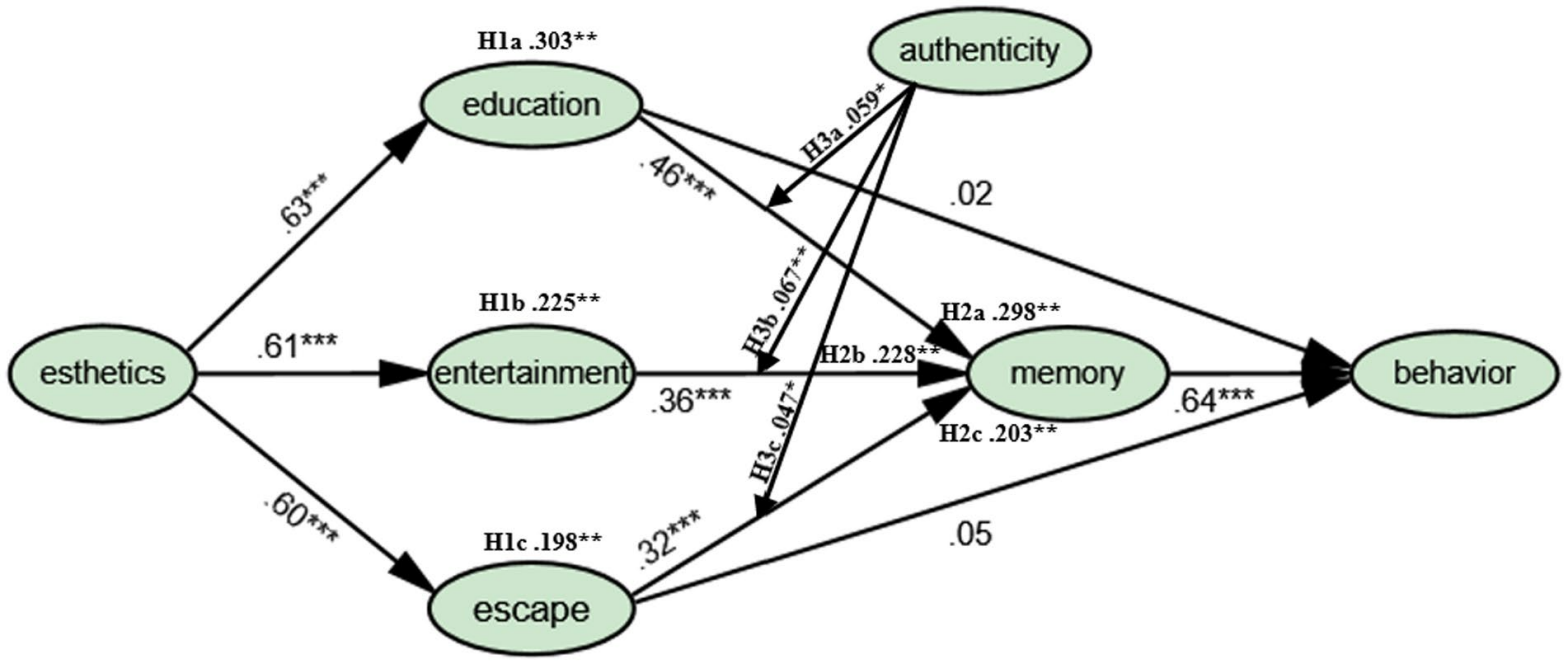
Estimate results of the model. *P < 0.05, **P < 0.01, ***P < 0.001.

Amos’s maximum likelihood method is used to test each hypothesis of the theoretical model. The model fit is basically in line with the standards recommended by scholars. GFI, NFI, RFI, CFI, IFI, and TLI are all greater than the recommended value of 0.9. AGFI = 0.865 and RFI = 0.893 are slightly lower than 0.9. RMSEA = 0.063, slightly higher than the recommended value of 0.5. F. Chen et al. (2008) specifically studied RMSEA and concluded that RMSEAs below 0.08 are acceptable. In other words, these values are within the acceptable range. Table 2-Structure presents the detailed results. As shown in Figure 3, all the direct influence paths are supported, apart from the direct effect of the experience economy (education: β = 0.02, entertainment: β = 0.04, and escape: β = 0.05) on behavior.

### Mediating effect

The Bootstrap (Bootstrap = 2000) method is used to detect the mediating hypothesis made above. Table 4 shows that all mediating effects exist. In other words, in addition to the direct impact of esthetic experience on memory, esthetic experience also has an indirect effect on memory through educational experience, entertainment experience, and escape experience. Because educational experience, entertainment experience, and escape experience have no direct effect on behavioral intention, memory plays a complete mediating role in the influence of educational experience, entertainment experience, and escape experience on behavioral intention. The influence coefficients are: H2a = 0.298, H2b = 0.228, H2c = 0.203; see Figure 3 and Table 4.

**Table 4:** Mediation effect.

Hypothesis		Point estimate	Bias-corrected 95%CI		Percentile 95%CI		Mediator
			Lower	Upper	Lower	Upper	
H1a	Esthetics →Education →Memory	0.303**	0.164	0.563	0.164	0.557	Yes
H1b	Esthetics →Entertainment →Memory	0.225**	0.120	0.447	0.116	0.439	Yes
H1c	Esthetics →Escape →Memory	0.198**	0.095	0.367	0.092	0.364	Yes
H2a	Education→ Mem → Behavior	0.298**	0.111	0.739	0.113	0.751	Yes
H2b	Entertainment → Mem → Behavior	0.228**	0.069	0.482	0.081	0.545	Yes
H2c	Escape → Mem → Behavior	0.203**	0.057	0.506	0.060	0.531	Yes

*P < 0.05, **P < 0.01, ***P < 0.001.

### Moderating effect

In this study, the Process V31 module was used to verify the moderating effect of the model, and control variables such as gender, age, education level, tourist type, and income were added to the analysis. Model 2 in Table 5 shows that hypothesis H3a is supported; that is, authenticity has a positive moderating effect on the influence of education on memory (β = 0.059, *t* = 2.528, *p* < 0.05). Model 4 confirms that hypothesis H3b is also supported; that is, authenticity has a positive moderating effect on the influence of entertainment on memory (β = 0.067, *t* = 3.194, *p* < 0.01). Model 6 confirms that hypothesis H3c is also supported; that is, authenticity has a positive moderating effect on the influence of escape on memory (β = 0.047, *t* = 2.122, *p* < 0.05).

**Table 5:** The moderating effects of authenticity.

Dependent variables	Memory											
	Model 1		Model 2		Model 3		Model 4		Model 5		Model 6	
	Coef.	*t*	Coef.	*t*	Coef.	*t*	Coef.	*t*	Coef.	*t*	Coef.	*t*
Interrupt	1.232	4.149	5.054	21.359	1.91	6.251	4.769	19.02	1.632	5.521	4.861	20.09
Control variables												
Gender	0.157	2.068	0.137	1.806	0.244	3.002	0.228	2.839	0.214	2.751	0.211	2.736
Age	0.049	1.627	0.046	1.522	0.041	1.25	0.039	1.23	0.029	0.946	0.024	0.777
Education	0.028	0.801	0.021	0.597	0.031	0.802	0.021	0.565	0.020	0.536	0.140	0.386
Tourism type	0.082	1.125	0.09	1.237	0.142	1.804	0.136	1.751	0.141	1.876	0.139	1.865
Income/per month	−0.088	−2.48	−0.088	−2.512	−0.062	−1.624	−0.062	−1.66	−0.061	−1.696	−0.057	−1.58
Authenticity (Aut)	0.183	6.803	0.177	6.609	0.126	3.956	0.151	4.66	0.169	6.006	0.173	6.169
Independent variable												
Education (Edu)	0.541	13.395	0.582	13.444								
Entertainment (Ent)					0.418	10.315	0.463	10.92				
Escape (ESC)									0.459	12.274	0.475	12.51
Moderator												
Edu * Aut			0.059	2.528								
Ent * Aut							0.067	3.194				
Esc * Aut											0.047	2.122
Model statistics												
R^2^	0.503		0.512		0.423		0.440		0.474		0.481	
R^2^ adj.	0.493		0.503		0.412		0.424		0.464		0.474	
F	50.542		45.704		36.675		34.21		45.078		40.401	

*n* = 358.

**Figure 4:**
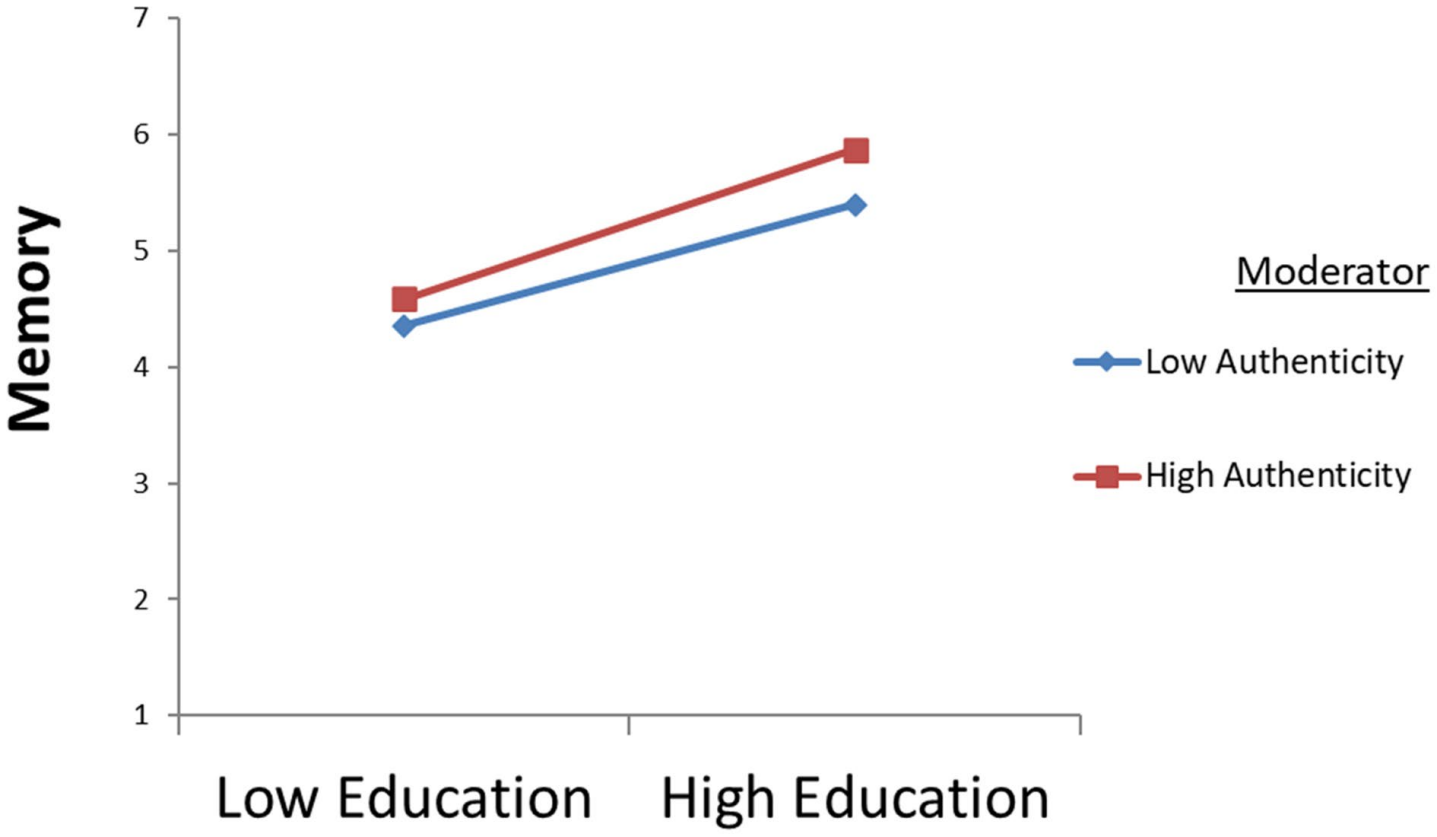
The moderating effects of authenticity on relationship between education and memory.

**Figure 5:**
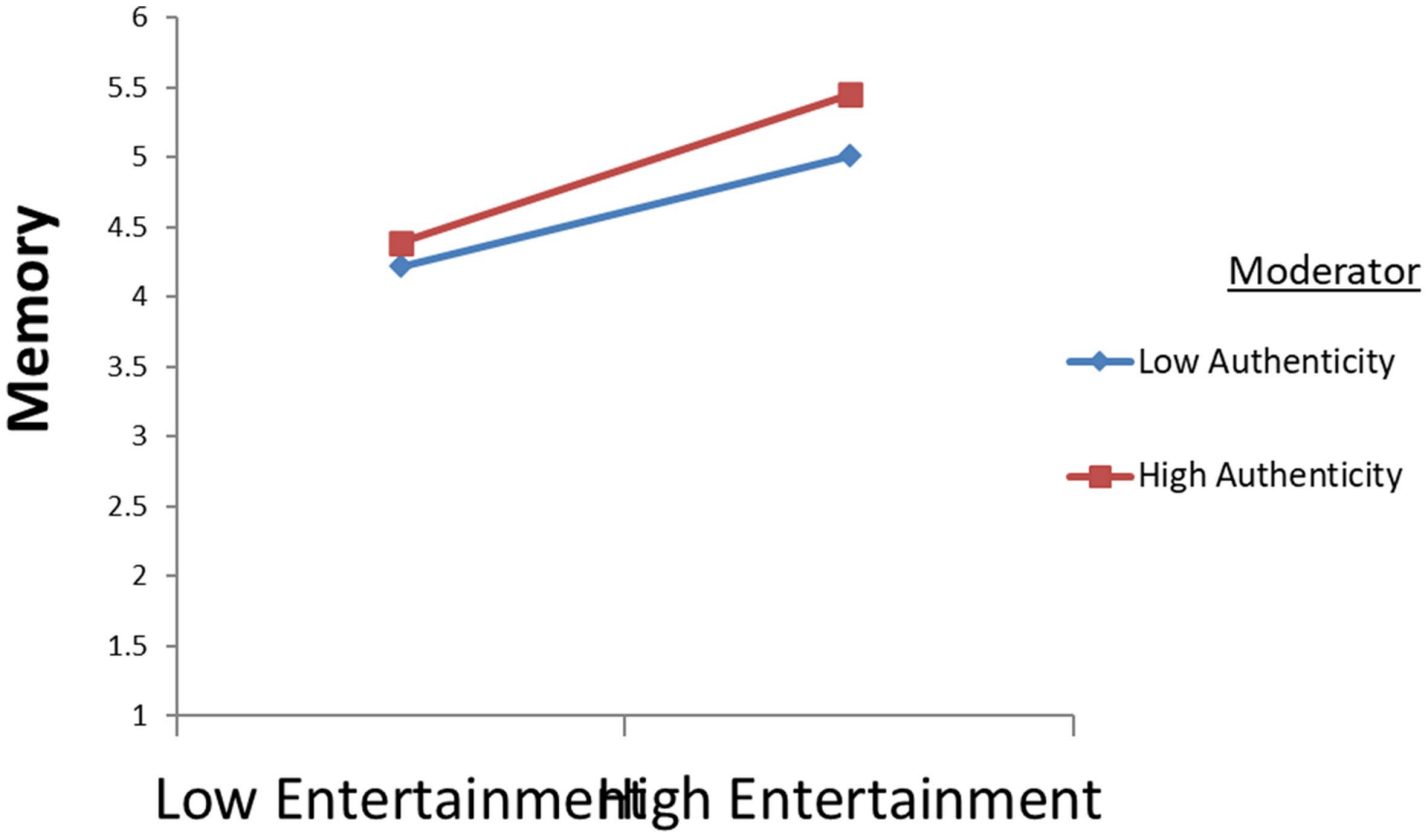
The moderating effects of authenticity on relationship between entertainment and memory.

**Figure 6:**
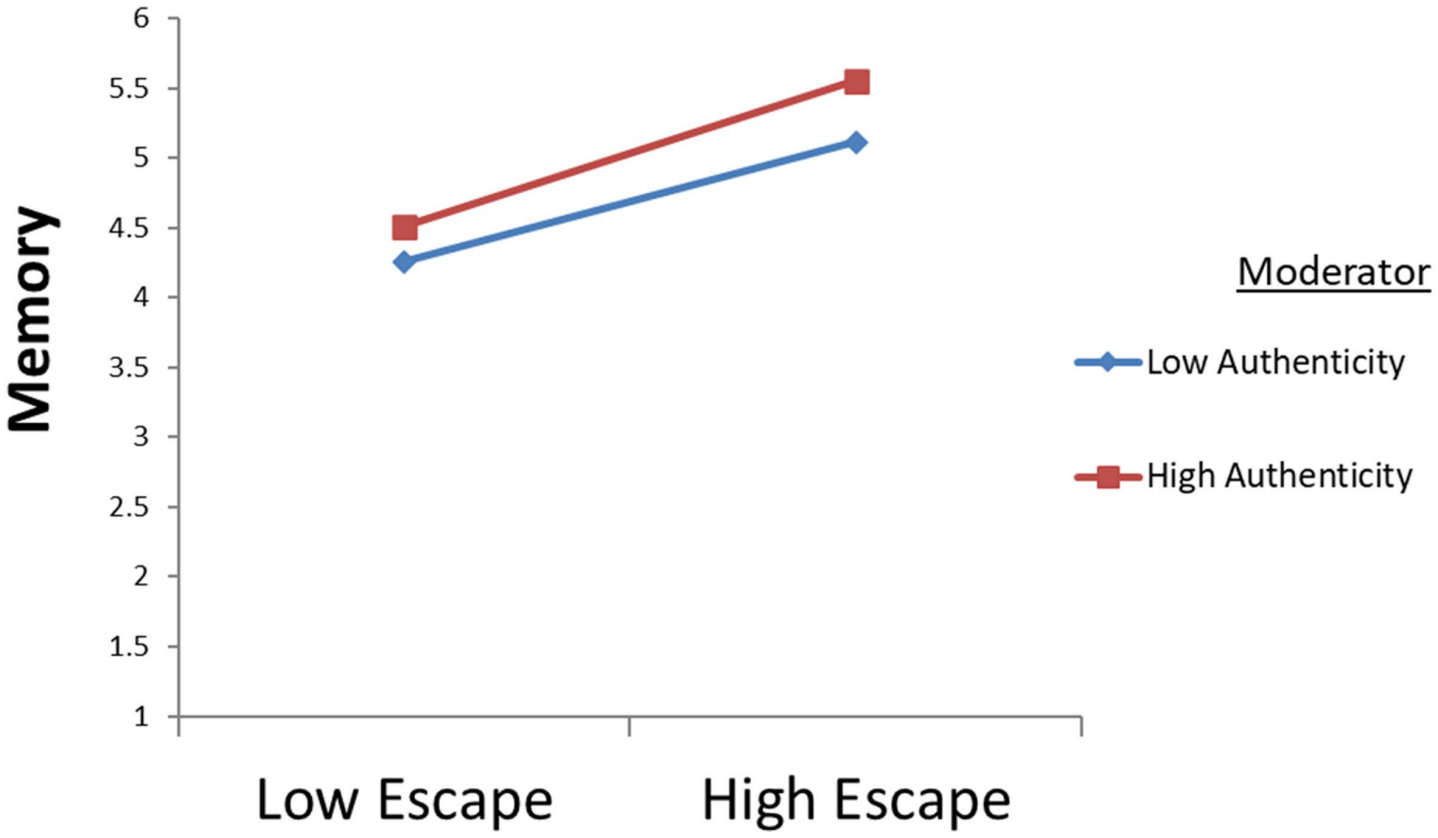
The moderating effects of authenticity on relationship between escape and memory.

This study uses curve analysis (Preacher et al., 2006) to reflect more intuitively the changes in authenticity and the changes in the relationship between education and memory, entertainment and memory, and escape and memory; see Figures 4–6. The results show that when authenticity is stronger, the effect of education on memory is stronger; when authenticity is stronger, the effect of entertainment on memory is stronger; similarly, the stronger the authenticity, the stronger the influence of escape on memory. The above findings suggest that tourists’ perception of authenticity will significantly moderate the relationship between education and memory, the relationship between entertainment and memory, and the relationship between escape and memory (All hypothesis’ T–Value >1.96).

## Discussion, conclusion, and contribution

### Discussion

The results of empirical analysis show that in the experience economy, esthetics is the antecedent variable of education, entertainment, and escape (Lai et al., 2020). This is consistent with the findings of Tom Dieck et al. (2018), indicating that esthetics has the most important position in the experience economy. This result also validates the findings of Hosany and Witham (2010), Mykletun and Rumba (2014), and Oh et al. (2007). The other three variables of the experience economy, education, entertainment, and escape all have a positive effect on memory. This result is similar to the findings of Oh et al. (2007), Kastenholz et al. (2018), and Tom Dieck et al. (2018). In addition, as the results of the mediation test show, esthetic experience also indirectly affects memory through education, entertainment, and escape, indicating that education, entertainment, and escape all contribute to enhancing the esthetic experience of tourists and the formation of more powerful memories.

However, as the results of the analysis show, in the experience economy, education, entertainment, and escape have no direct effect on behavior. The results of this study are similar to the results of Zhang et al. (2021). Their study found that the four dimensions of the experience economy had no direct effect on revisit intention. Research by Ballantyne et al. (2011) found that the experience economy, as an external stimulus, can arouse specific emotions in tourists, affecting their behavioral intentions. This result is similar to the findings of Lai et al. (2020) that the experience economy will affect the word-of-mouth of tourists through memory. However, word-of-mouth is also a behavioral intention. The results of this study confirm that the education, entertainment, and escape elements of the experience economy influence behavior through memory. That is, memory plays a fully mediating role in the influence of the experience economy on behavioral intentions.

Another important result of this study is that authenticity plays a positive moderating effect on the influence of the experience economy on memory; that is, tourists’ perception of authenticity reinforces the impact of education, entertainment, and escape on memory. This result confirms research by Manthiou et al. (2018), who argued that our work highlights the need of investigating authenticity as a theoretical construct in the experience economy because of its connection with mental influences such as memory. In their study, authenticity has a direct effect on memory. Zatori et al. (2018) found that experience has a direct effect on authenticity. Manthiou et al. (2014) found that experience has a direct effect on memory. Wang et al. (2020) also found that the experience economy has a direct effect on authenticity and memory. Based on previous research results, we believe that the three moderating effects of authenticity in the experience economy are credible and that this constitutes a valuable discovery.

### Theoretical contributions

The following five elements constitute the theoretical contributions of this research: (1) Introducing the experience economy variables into ICH tourism has expanded the application scope of experience economy theory; (2) Adopting the new experience economy structure of Tom Dieck et al. (2018), verifying the structure, and reconfirming the rationality of the structure; (3) Finding that education, entertainment, and escape have a mediating effect in the relationship between esthetics and memory and that education and escape have a mediating effect in the relationship between esthetics and authenticity; (4) Finding that memory plays a complete mediating role between experience economy and behavioral intention; and (5) Finding that authenticity plays a positive moderating role in the relationship between experience economy and memory.

Although experience quality has been studied in ICH tourism (López-Guzmán and Santa-Cruz, 2017; Su et al., 2020), the concepts of the experience economy and of experience quality are different. The experience economy is another type of economy following on from the agricultural, industrial, and service economies. “Experience” will become an independent economic output and the fourth economic offering after “products,” “commodities,” and “services,” and will serve as a “new source of value” in the future economy (Pine and Gilmore, 1998; Pine et al., 1999). Experience quality refers to an internal evaluation by the customer after an experience. This evaluation is not objective, and everyone’s experience is not consistent (Lemke et al., 2011). ICH tourism is the most important type of cultural tourism (Su et al., 2019). It constitutes a category of the experience economy. Therefore, it is essential to introduce experience economic variables into ICH tourism.

In previous studies, the four variables of the experience economy all appeared as a second-order whole or in the form of juxtaposition. At the same time, Tom Dieck et al. (2018) searched previous research and found that esthetics are often the most important elements in the relevant studies of the experience economy. Based on this finding, they proposed a new structure, that is, that esthetics is the antecedent variable of education, entertainment, and escape, and is verified by the VR experience. This study adopted this new structure and used it to explore the relationship between the experience economy and memory, authenticity, and behavioral intention. The results confirm that Tom Dieck et al. (2018) research framework is reasonable. However, in our study framework, the relationship between the experience economy and authenticity is not consistent with previous research results. In previous research, the experience economy emerged as a second-order structure, and the relationship between each sub-dimension and authenticity was explored separately. The results of this study that authenticity plays a positive moderating role in the relationship between education and memory, entertainment and memory, and escape and memory, a role which was not apparent in the original structure. This is where our structure is superior to the original structure, and it represents an important finding of this study.

In Tom Dieck et al. (2018) study, the authors confirmed the direct impact of aesthetics on education, entertainment, and escape. There was no further in-depth discussion on whether education, entertainment, and escape play a mediating effect in esthetics and other outcome variables. However, judging from the results of the present study, such a mediating effect exists. Our discovery goes further than the findings of Tom Dieck et al. (2018), and it serves as a reference for other scholars in future research.

### Practical implications

The results of this study show that esthetics is the most important factor in the experience economy, and it is the important element whereby tourists make good memories and perceptions of authenticity, thereby generating revisits, recommendations, and loyalty behaviors. Therefore, in tourism development, we must pay attention to highlighting the aesthetic elements of ICH. Pine and Gilmore (1998) outline of the experience economy is mainly based on two dimensions: passive to active participation and desire from absorption to immersion. Esthetics is a passive desire for participation and immersion. Only by enhancing the effect of esthetics can the interactive and immersive experience of tourists be achieved.

Second, education, entertainment, and escape all have a positive effect on memory, and education, entertainment, and escape play a mediating role in the relationship between esthetics and memory. Therefore, in product development and design, education should be based on esthetics and entertainment, and the function of entertainment should be strengthened, thereby generating escape emotions and enhancing the positive memories formed by tourists. For example, in the tourist experience, the frequency of product explanations could be increased. A good experience atmosphere could be created by using, for example, VR (Virtual Reality) and AR (Augmented Reality) equipment to strengthen the experience immersion of tourists.

Third, memory plays an important role in the behavioral intention of ICH tourists. Therefore, creating good memories is the top priority in the marketing process of the tourism industry. Good memories cause tourists to have subsequent behavioral intentions. In the tourists’ experiences, we can strengthen their memories by using scientific and technological means, publicity frequency, and bright color stimulation.

Fourth, authenticity plays a positive moderator role in the relationship between the experience economy and memory. It strengthens the effect of education, entertainment, and escape on memory. Therefore, in the process of ICH tourism development, attention should be paid to the cultural attributes of the heritage itself to strengthen the authenticity perception of tourists, as emphasized in previous studies.

Finally, in the protection practice of ICH, non-profit organizations such as museums, art galleries, and concert halls can create a good experience atmosphere through innovative advertising and education to strengthen the role of education and escape so that tourists can perceive authenticity. The main function of these organizations is to expand the knowledge of tourists and to obtain social benefits in addition to economic benefits.

## Research limitations and future study

First, the research object of this study is the performance ICH project. However, as there are many types of ICH with different attributes, caution should be used in expanding the research results to other ICH experiences. Second, the data in this study are cross-sectional, and the results of different investigation times could be slightly different. Third, the survey took place during the long holidays of “Golden Week.” Most of the tourists were on group tours. Therefore, our results may not cover all types of tourists.

Future research could use different research methods, such as qualitative research methods to conduct in-depth interviews with tourists, and then use the grounded theory method to encode, refine the research results and arrive at different research results. In addition, there are other important variables in the marketing process of tourism enterprises, such as value perception, satisfaction, involvement, and engagement. Subsequent research could appropriately introduce other variables to carry out similar research and explore the differences in results to provide a more valuable reference for tourism enterprises.

## Data availability statement

The original contributions presented in the study are included in the article/supplementary material, further inquiries can be directed to the corresponding author.

## Author contributions

YC, JN, and YT conceived the study. YC, TM, and YT wrote the manuscript. All authors designed the study, collected and analyzed the data, read and approved the manuscript, and agreed to be accountable for all aspects of the work.

## Conflict of interest

The authors declare that the research was conducted in the absence of any commercial or financial relationships that could be construed as a potential conflict of interest.

## Publisher’s note

All claims expressed in this article are solely those of the authors and do not necessarily represent those of their affiliated organizations, or those of the publisher, the editors and the reviewers. Any product that may be evaluated in this article, or claim that may be made by its manufacturer, is not guaranteed or endorsed by the publisher.
